# Challenges in studying air pollution to neurodegenerative diseases

**DOI:** 10.1016/j.envres.2025.121597

**Published:** 2025-04-11

**Authors:** Dayoon Kwon, Kimberly C. Paul, Karl O’Sharkey, Seung-A Paik, Yu Yu, Jeff M. Bronstein, Beate Ritz

**Affiliations:** a Department of Epidemiology, Fielding School of Public Health, University of California, Los Angeles, United States; b Department of Neurology, David Geffen School of Medicine, University of California, Los Angeles, United States; c Center for Health Policy Research, Fielding School of Public Health, University of California, Los Angeles, United States

**Keywords:** Air pollution, Neurodegenerative diseases, Alzheimer’s disease and related dementias, Parkinson’s disease

## Abstract

Exposure to ambient air pollution is ubiquitous and unavoidable. While associations between air pollution and cardiometabolic diseases are well-established, its role in neurodegenerative diseases, such as Alzheimer’s disease and related dementias (ADRD) and Parkinson’s disease (PD), has only recently begun to emerge. This narrative review provides an overview of current findings and discusses challenges and opportunities for future epidemiologic research. Mechanistically, air pollution may contribute to ADRD and PD through neuroinflammation, oxidative stress, and cerebrovascular damage. Long-term exposure to high levels of air pollution may increase the risk of ADRD and PD. Over the past 20 years, more than 50 studies have examined air pollution and ADRD, while fewer studies have focused on PD. Although the estimated effects are modest in size, they translate into a substantial number of affected individuals due to the widespread nature of the exposure and an increasingly aging population worldwide. Future research should extend exposure periods to cover younger and middle ages, estimate the effects of long-term cumulative exposures, and evaluate moderators and mediators, such as diet, physical activity, green space, and noise. More studies are also needed to include large and diverse populations, including those with special vulnerabilities and emerging exposures like wildfire smoke.

## 1. Introduction

Air pollution is the leading risk factor for premature death worldwide (State of Global Air, 2019). Long-term exposure to particulate matter (PM_2.5_) contributed to 4.1 million deaths and 118 million disability-adjusted life years in 2019, primarily due to cardiovascular and respiratory diseases (State of Global Air, 2019). However, widely recognized global health assessments often overlook the impact of air pollution on neurodegenerative disorders despite growing evidence of its harmful effects on the brain and plausible mechanisms such as excessive oxidative stress and neuroinflammation (Block and Calderon-Garciduenas, 2009; Jayaraj et al., 2017).

The biological mechanisms through which air pollution affects brain health are not fully understood, but several pathways are plausible. Ultrafine particles (UFP) and constituents of particulates can adsorb toxic compounds on their surfaces. These particles are inhalable and can reach the brain via the bloodstream and/or by direct diffusion through the olfactory nerve or nasal mucosa. Exposure to air pollution can induce systemic inflammation, damage the blood-brain barrier, and activate microglia (Block and Calderon-Garciduenas, 2009). Additionally, once inhaled these particles may indirectly affect brain health by triggering the release of inflammatory cytokines or other chemicals from the lungs (Jayaraj et al., 2017).

Here, we discuss some challenges of studying the association between air pollution and neurodegenerative diseases, particularly Alzheimer’s disease and related dementias (ADRD) and Parkinson’s disease (PD), which are the most prevalent neurodegenerative disorders, affecting 52 million and 9 million individuals worldwide in 2019, respectively (State of Global Air, 2019). As these disorders progress with age and populations are aging, they increasingly contribute to economic and social burdens through direct medical costs and the demands for formal and informal care.

### 1.1. Air pollution and neurodegenerative diseases

In recent years, a growing body of epidemiological studies has begun to explore the potential link between air pollution and neurodegenerative diseases. Yet, challenges persist, including varying exposure contrasts, levels, and duration, as well as complexities involving co-exposures and different pollution sources. Key issues also include determining the most relevant latency periods for neurodegenerative diseases, diagnostic accuracy, and understanding the impacts of mediators such as comorbidities.

Despite these complexities, meta-analyses conducted within the last five years have consistently shown that higher levels of PM_2.5_ increase the risk of AD and PD (Table 1). Specifically, for every 10 μg/m^3^ increase in PM_2.5_, the relative risk of AD has been estimated to increase by 1.5- to 5-fold, while the risk of PD increases somewhat less strongly, with meta-analytical effect estimates currently ranging from 1.06 to 1.34. A meta-analysis by Tsai et al. (2019) reported the highest hazard ratio (HR) of 4.82 (95 % confidence interval [CI] = 2.28–7.36) for PM_2.5_ exposure and AD. The analysis included data from only three studies with region-specific high effect estimates: the United Kingdom (HR = 2.88, 95 % CI = 1.25–6.29), the United States (HR = 4.05, 95 % CI = 2.84–5.69), and Taiwan (HR = 7.08, 95 % CI = 5.99–8.35). Given the small number of included studies, the generalizability of this finding is limited. This result underscores not only the variability in the PM_2.5_ effect estimates across studies that may be explained by differences in study methodologies or populations but also the importance of carefully assessing inclusion criteria for study results that are summerized in meta-analyses.

Associations between nitrogen dioxide (NO_2_) and ground-level ozone (O_3_) and these neurodegenerative disorders are less consistently reported. While some meta-analyses report no associations, others have found modest positive associations that did not reach formal statistical significance (Table 1). Even small or modest size increases in disease risk for these air pollutants and neurodegenerative diseases can have major public health implications, as air pollution is ubiquitous in nature and difficult to avoid due to its widespread presence in the environment. While mitigation efforts can reduce exposure, the global and pervasive nature of air pollution means that populations remain continuously exposed, potentially amplifying the overall health burden in an aging human population. The major remaining question, however, is whether NO_2_ and O_3_ affect risk independently of fine PM or may even exacerbate the risk. This question is particularly relevant as both PM_2.5_ and NO_2_ levels have been reduced considerably in the USA and Europe, while controlling O_3_ levels has proven more difficult, resulting in relatively stable levels (Lv et al., 2021). O_3_ is not directly emitted but results from secondary photochemical reactions influenced by volatile organic compounds (VOCs) and other pollutants, and its levels depend on meteorological conditions. Additionally, it is important to note that modeling O_3_ and NO_2_ often creates collinearity (negative correlations) due to the modeled atmospheric reactions involving both O_3_ and NO_2_. This underscores the importance of exploring O_3_’s independent effects as much as possible. Future studies might need to focus on regions where O_3_ is elevated due to atmospheric transport rather than local emissions. Such areas, often characterized by large green spaces with few pollution sources and low population density, would have high O_3_ but low NO_2_ levels. Conversely, urban areas with heavy traffic and dense populations tend to have high NO_2_ and lower O_3_ due to titration effects (Yang et al., 2018).

### 1.2. Exposure assessment issues

To assess the contributions of air pollution exposures to neurodegenerative diseases, which are likely caused by chronic neurotoxicity and damage that slowly accumulates over time, it is necessary to investigate these disorders in large populations. This requires decades of air monitoring and detailed address history data. Large and long-running longitudinal cohort studies (such as the Nurses’ Health study) or countries with residence records, a universal health insurance system, and routine air monitoring systems – like Scandinavian or some Asian countries (Korea and Taiwan) – provide currently some of the best available research settings. Although direct measurements from air quality monitors are generally very accurate in terms of temporal variation, they may not reflect exposure over larger geographical areas if local sources affect air pollutant concentrations and their variability at a scale not supported by the density of monitoring sites. Studies have addressed some of these issues by building land-use regression or emissions-based models with high spatial resolution to capture transportation-related exposures and using various modeling techniques to incorporate data from satellites, ground monitors, and meteorological sources (Meng et al., 2019; Wu et al., 2016). However, modeled exposure surfaces often provide high resolution only in one of the two dimensions, i.e., either in terms of temporal or spatial coverage. Ambient air pollutant exposure monitoring and modeling limitations are most likely contributing to non-differential exposure misclassification, which can be extensive for pollutants with high spatial variability, such as traffic pollutants like NO_2_ and certain source-specific components of fine particles (e.g., UFP, fresh exhaust, and brake and tire wear-related metals), potentially biasing study results toward the null.

The most sophisticated air pollution models and the densest monitoring for many pollutants have been developed after the year 2000. This limits our current ability to understand the long-term contributions of air pollutants to the neurodegenerative process, which likely starts many decades before diagnosis or during the long prodromal stages of the disease. In fact, most air pollution studies assessing ADRD and PD have only evaluated exposure over periods ranging from one to five years prior to diagnosis, which is insufficient to capture cumulative effects and does not take prolonged prodromal periods into consideration. Research covering longer periods is necessary to appropriately address lifetime cumulative exposure effects and identify the most vulnerable periods for the neurodegenerative process. Detailed address history data allow for the investigation of time-varying exposures, including changes in residence, but are not consistently available or integrated into exposure models. As a result, exposure measures employed in these large studies rarely take into account changes in residences, do not assess ambient exposures at work addresses, and do not employ time-activity data, thus solely relying on estimating outdoor air pollution exposure at a single address or over a short period of time.

The role of different air pollution sources and the challenge of distinguishing effects of components from exposure mixtures further complicate air pollution studies. This is also important in order to understand inconsistencies between studies better, as the composition and sources of PM_2.5_ mass can vary over time and across locations, likely affecting the toxicity of a PM_2.5_ mass-based measure. Most studies to date have not considered variability in PM_2.5_ constituents and/or source components. Recent research conducted in the Environmental Predictors of Cognitive Health and Aging (EPOCH) cohort with data from the Health and Retirement Study (HRS) reported that higher concentrations of modeled PM_2.5_ averaged over a 10-year period – generated by agricultural burning and wildfires – were associated with greater rates of incident dementia. Specifically, for each interquartile range increase in PM_2.5_, the HR was 1.13 (95 % CI = 1.01–1.27) for agriculture and 1.05 (95 % CI = 1.02–1.08) for wildfires (Zhang et al., 2023). These findings underscore the importance of considering source-specific PM_2.5_ contributions (e.g., using biomass burning markers) to ADRD and PD.

Moreover, challenges persist in disaggregating the effects of individual air pollutants. For example, NO_2_, often used as a marker of traffic-related air pollution, and O_3_, a secondary pollutant formed from reactions between nitrogen oxides and VOCs in the presence of sunlight, exhibit sharp concentration gradients near traffic sources. Although photochemical reactions can lead to O_3_ formation, rapid titration reactions reduce O_3_ concentrations in areas with high NO_2_ levels, particularly in locations with high nitrogen oxide emissions, such as near traffic sources. These dynamics may necessitate using multi-pollutant or mixture models to accurately assess the individual and combined impacts of pollutants on neurodegenerative conditions. Additionally, addressing air pollution contributions may be complicated by correlated co-exposure to other environmental exposures that might act as confounders or modifiers, such as lack of green space and higher traffic-related noise.

While most studies focus on outdoor air pollution and its links to ADRD and PD, emerging evidence suggests that indoor air pollution may also contribute to neurodegeneration (Li et al., 2020; Zheng et al., 2022). Indoor sources such as cooking, secondhand smoke, and household products release PM_2.5_, NO_2_, and VOCs that have not been sufficiently addressed yet (Chen et al., 2023). Given that some older adults spend much of their time indoors, integrating indoor air quality assessments into future studies may be especially important for studies concerned with the progression of neurodegenerative diseases. However, comprehensive indoor air quality assessments currently still require expensive equipment and long sampling durations, which limits their feasibility in large-scale population studies (Tryner et al., 2021). Mitigation strategies, including improved ventilation, cleaner cooking and heating fuels, and reduced use of high-emission household products, may not only reduce exposures but could be employed in intervention studies conducted in indoor environments with elderly at-risk populations.

Apart from methodological challenges, environmental justice considerations are essential when evaluating the impact of air pollution on neurodegenerative diseases. Neighborhood-level disparities influence both exposure levels and susceptibility to adverse health effects. Low-income and marginalized communities often experience higher air pollution burdens due to residential proximity to highways, industrial zones, and other pollution sources, as well as limited access to healthcare and preventive services (Adhikari et al., 2024). These factors may also exacerbate disparities in neurodegenerative disease risk. Addressing environmental justice in air pollution research requires a multidisciplinary approach, integrating social determinants of health into epidemiological models and advocating for policies that reduce exposure inequities.

### 1.3. Diagnostic accuracy, latency, and relevant time periods

Given the complexity of symptoms and pathomechanisms, accurately identifying the onset of neurodegenerative disorders presents substantial challenges in epidemiological studies, especially when considering the long-term impacts of air pollution. AD is the most common form of ADRD, accounting for about 60–70 % of dementia cases (World Health Organization, 2023). It is characterized by progressive cognitive deterioration, typically beginning with short-term memory loss, and a definitive diagnosis currently only possible at autopsy. Most cases of ADRD exhibit a mixed pathology, often including vascular components alongside AD pathologies. They may also display pathologies associated with PD, such as those seen in dementia with Lewy bodies. PD, primarily a movement disorder, exhibits a prodromal period where non-motor symptoms (e.g., olfactory dysfunction, rapid eye movement sleep behavior disorders, constipation, and depression) may manifest up to a decade before motor symptoms appear. Similarly, Lewy body dementias, which include Parkinson’s disease dementia (PDD) and dementia with Lewy bodies (DLB), present a mix of cognitive and motor symptoms. About 80 % of PD patients who survive for 20 years will eventually develop PDD (Hely et al., 2008). In cases of DLB, cognitive symptoms either precede or emerge within one year of parkinsonism, which is a requirement for clinical diagnosis. The distinction between DLB and PD in clinical practice can be challenging. Pathologically, many patients diagnosed with PD during their lifetime may exhibit signs of DLB upon autopsy. This overlap indicates that, while distinct in clinical criteria, PD and DLB share underlying pathological features.

Accurately diagnosing neurodegenerative diseases is inherently challenging due to the subtlety and variability of early symptoms and a lengthy prodromal period during which some individuals with better reserves may compensate for diminished cognitive or motor function. For example, research indicates that when compared with a diagnosis based on brain pathology, the diagnostic accuracy of a clinical diagnosis for PD varies, with non-experts achieving 73.8 % accuracy and movement disorder experts 83.9 % after a follow-up period (Rizzo et al., 2016). Further complicating epidemiological studies of disease incidence is an even higher rate of misdiagnosis early in disease (Rizzo et al., 2016). Currently, many cohort studies rely on interview surveys with participants self-reporting diagnoses or symptoms. The latter relies on respondents’ awareness of symptoms, which can be influenced by cultural/social factors and educational levels, while the timing of getting a first diagnosis depends on access to healthcare in the US. Similar misdiagnosis issues apply to large record-based studies using, for example, Medicare billing or hospitalization records to generate the outcome data. In addition, these studies have the added problem that patients with neurodegenerative diseases are generally being admitted for such a diagnosis in the late stages of the disease or are admitted to departments other than neurology for comorbidities that require hospitalization. These diagnostic difficulties profoundly impact studies relying on administrative records to examine the links between air pollution and disease incidence as they contribute to disease misclassification.

Consequently, relying solely on electronic hospitalization records to identify ADRD and PD is problematic, not just due to misdiagnosis but also because of the inherent difficulties in pinpointing the likely time of onset of disease. These records often capture diagnoses at advanced stages of the disease or are influenced by admissions for other comorbidities, obscuring the early stages of neurodegenerative diseases. Similarly, death certificates present additional challenges as they frequently underreported conditions like AD or PD, listing them as contributing rather than primary causes of death. The complexity of differentiating between the early and later stages of ADRD and the initial non-motor symptoms followed much later by the typical motor features of PD may contribute to inconsistencies in results reported in record-based studies of air pollution for these neurodegenerative diseases.

### 1.4. Comorbidities, lifestyle factors, and aged cohorts

Comorbidities such as cardiovascular disease (CVD) and diabetes may mediate the effects of air pollution on neurodegenerative disorders. Air pollution is a known risk factor for cardiovascular health and can trigger acute myocardial infarction (Mustafic et al., 2012) and stroke (Toubasi and Al-Sayegh, 2023). Because CVD accelerates cognitive decline and can lead to neurodegenerative conditions, exposure to air pollution might negatively affect cognition, even without directly affecting the brain, through the detrimental effect of CVD on brain health. Notably, higher levels of the 5-year average modeled total PM_2.5_ were shown to be associated with an increased hazard of developing vascular dementia (HR = 1.66 per 0.88 μg/m^3^ of PM_2.5_ increase, 95 % CI = 1.38–1.99), supporting the role of air pollution on the vascular component of ADRD that may further aggravate amyloid driven angio-pathies and contribute to mixed pathologies (Grande et al., 2020). Recent studies suggest that these effects are mediated by air pollution’s impact on cardiovascular health rather than CVD acting as a moderator (Grande et al., 2020; Paul et al., 2020).

Epidemiological evidence indicates that air pollution is also a risk factor for diabetes (Weinmayr et al., 2015), which in turn is a strong risk factor for ADRD (Ohara et al., 2011). Type 2 diabetes mellitus (T2DM) shares many similarities with AD, such as impaired glucose uptake, increased oxidative stress, inflammation, and brain atrophy. Both conditions can lead to impaired cognition and dementia. Moreover, T2DM has been directly linked to brain insulin resistance and amyloid accumulation in AD, underscoring a more direct connection beyond inflammation (Paul et al., 2018; Stanciu et al., 2020). While T2DM has also been associated with an increased risk of developing PD (Yue et al., 2016), the evidence linking diabetes and PD is less consistent compared to diabetes and AD (Han et al., 2023; Palacios et al., 2011). Given the strong links between air pollution, diabetes, and ADRD and possible also with PD, the role of CVD and diabetes as mediators in these relationships must be carefully considered. Importantly, adjusting for these comorbidities in studies may be inappropriate since they may be in the pathways and explain a substantial proportion of air pollution-related neurodegenerative diseases. This also suggests that exposure periods of high relevance might be even earlier in life, i.e., prior to the onset of these common comorbidities.

Similarly, depression may act as a partial intermediate in the association between air pollution and neurodegenerative diseases. Air pollution-induced neuroinflammation, oxidative stress, and blood-brain barrier disruption are known contributors to both depression and neurodegeneration (Qiu et al., 2023; Roy and D’Angiulli, 2024). Depression itself has been linked to neurotoxic processes such as hypothalamic-pituitary-adrenal-axis dysregulation and reduced neurogenesis, which may accelerate ADRD and PD progression (Qiu et al., 2023; Roy and D’Angiulli, 2024). Recent epidemiological studies suggest that depression mediates a portion of the air pollution-cognitive decline association (Qiu et al., 2023; Wang et al., 2025), though air pollution also exerts direct neurotoxic effects on amyloid-beta/tau accumulation (ADRD) and dopaminergic neurons (PD), independent of depression (Ai et al., 2024; Krzyzanowski et al., 2024). Given the interplay between air pollution, depression, and neurodegeneration, studies should carefully consider its role and avoid overadjustment in statistical models and underestimation of the total effect of air pollution on ADRD and PD.

As for these comorbidities, lifestyle factors such as diet and physical activity may modify the relationship between air pollution and neurodegenerative disorders. Healthy dietary habits reduce the risk of major noncommunicable diseases, including CVD (Lim et al., 2019), diabetes (Rasmussen et al., 2020), ADRD (Ellouze et al., 2023), and PD (Kwon et al., 2023). A healthy diet rich in antioxidants and anti-inflammatory nutrients can mitigate the chronic systemic inflammation that exacerbates the adverse effects of air pollution on cardiometabolic and neurodegenerative diseases. Specifically, the Mediterranean diet is associated with a lower mortality risk from PM_2.5_ and NO_2_-related CVD, possibly through suppressing arrhythmias, modulating autonomic function, and anti-thrombotic, anti-inflammatory, and vasodilatory effects (Lim et al., 2019). Moreover, vegetables and fruits, rich in nutrients like polyphenols, antioxidant vitamins, and dietary fiber, can reduce inflammation and oxidative stress caused by ambient pollutants in the central nervous system, ultimately affecting the pathogenesis of neurodegenerative disorders (Zhu et al., 2022). Therefore, it is prudent to consider the modifying effects of diet when assessing the impact of air pollution on neurodegenerative disorders, especially given its close association with comorbidities such as CVD and diabetes.

Physical activity also reduces the risk of major noncommunicable diseases, including cardiometabolic and neurodegenerative diseases (Pedersen and Saltin, 2015). The benefits of physical activity include decreasing lipid levels and inflammatory markers in circulation, promoting both cardiometabolic and brain health, and enhancing brain plasticity and cognitive reserve. Additionally, access to green spaces has been shown to increase physical activity among older populations, further reducing the risk of ADRD (de Keijzer et al., 2020). However, there are concerns about the potential risks of exercising in polluted areas. A recent systematic review has indicated that increased inhalation of air pollutants during physical activity can diminish its beneficial effects on the cardiovascular and metabolic systems, though findings on brain function remain inconsistent. For instance, brain-derived neurotrophic factor, a key neuroprotective marker, did not show differences when cycling was performed in higher versus lower-polluted environments (Chandia-Poblete et al., 2022). Thus, the advantages of physical activity for brain health must be carefully weighed against the environmental conditions of the exercise setting, particularly the proximity to traffic and urban pollution during exercise.

Beyond modifiable lifestyle factors, gene-environment interactions play a role in shaping individual susceptibility to air pollution’s neurotoxic effects. While air pollution is an emerging risk factor for neurodegeneration, individual genetic differences may determine the extent to which pollutants accelerate disease progression. The Women’s Health Initiative Memory Study found that women living in areas with elevated PM_2.5_ levels have an increased risk of cognitive decline and dementia, with the effect being particularly pronounced in Apolipo-protein E ε4/ε4 carriers (Cacciottolo et al., 2017). Although some monogenic mutations can directly cause familial forms of neurodegenerative diseases, most are polygenic and arise from complex gene-environment interactions rather than a single genetic determinant. Beyond individual genetic variants, emerging research on polygenic risk scores suggests that individuals with a high cumulative genetic risk for ADRD or PD may experience greater susceptibility to air pollution’s neurotoxic effects (Essers et al., 2023; Kwon et al., 2025). Polygenic risk score models aggregate multiple genetic risk variants into a single measure and provide a more comprehensive approach to identifying subpopulations at heightened risk. However, gene-environment interactions remain underexplored in air pollution epidemiology, with few studies incorporating genetic risk profiling into analyses of pollution exposure. Future research that aims to integrate genetic data into epidemiologic studies may be able to better characterize susceptible subpopulations and elucidate the biological mechanisms linking air pollution, genetic predisposition, and neurodegeneration. By incorporating gene-environment interaction frameworks, studies can improve risk stratification and enhance our understanding of how environmental and genetic factors jointly shape neurogenerative disease trajectories.

Finally, the selection of participants, especially in studies involving older cohorts, can substantially affect epidemiological research findings, as they typically include individuals over 50 years of age as shown in recent meta-analyses (Table 1). Factors such as baseline health status and attrition rates are particularly influential. Older adults who elect to participate in long-term studies might not represent the general elderly population as they tend to be healthier, more engaged, and possibly more conscientious about their health (Woods et al., 2016). This can cause selection bias based on under-enrollment of those with poorer health or less access to healthcare, and may bias results towards the null. Additionally, high attrition rates, often due to health deterioration from other chronic diseases related to air pollution that affect mobility, frailty, or mortality, can further contribute to selection bias and result in disproportionately removing sicker individuals who are more highly exposed and would develop the outcome from studies. This can also lead to an underestimation of air pollution impacts on neurodegenerative disorders, if those who remain in the study are healthier and less affected by air pollution on average. To mitigate these biases, researchers may, for example, want to consider inverse probability weighting to correct for differential dropout and missing data (Thompson and Arah, 2014). Moreover, close longitudinal tracking of participants can help researchers identify patterns of selective attrition, and this information may be used to adjust for biases. Ensuring that studies enroll diverse population samples, particularly encouraging participation of individuals from varying socioeconomic backgrounds and access to healthcare, can further enhance the generalizability and transportability of findings.

## 2. Conclusion

Studying the effect of air pollution on neurodegenerative diseases presents several notable challenges. The issues include limitations in exposure assessment, such as low exposure contrasts and short duration relative to the long latency periods of ADRD and PD. Additionally, co-exposure to other pollutants, environmental factors, and variations in PM_2.5_ constituents, sources, and toxicities add complexity. Accurate outcome assessment is also inherently challenging due to the subtlety and variability of early symptoms of ADRD and PD, complicating the understanding of disease onset and progression. Latency periods and identifying the most relevant time periods for exposure further complicate the assessment of the impact of air pollution. Mediators and/or effect modifiers (e.g., comorbidities and lifestyle factors) require careful consideration. Furthermore, the potential for selection bias in studies involving older cohorts at baseline should be carefully assessed or at least its potential magnitude estimated (Banack et al., 2019).

Thus, future research should focus on extending exposure assessment to include longer periods (greater than five years) to capture the cumulative effects and long-term impact of air pollution on ADRD and PD. Incorporating various risk mediators and/or modifiers, including comorbidities and behavioral factors, will help suggest mechanisms for the development of ADRD and PD. Additionally, including larger and more diverse study populations, particularly across different age groups, socioeconomic and racial/ethnic backgrounds, is essential to ensuring that the findings are generalizable to the broader aging population.

## Figures and Tables

**Table 1:** Results of meta-analyses investigating the association between long-term air pollution and neurodegenerative diseases.

First Author (Publication Year)	Number of studies included and time period	Country	Sample size and age group	Result
**Alzheimer’s disease**				
Gong et al. (2023)	13 studies 2015–2022	France, Hong Kong, Italy, Taiwan, the UK, and the US	168,414,337 individuals	PM_2.5_: OR = 1.65 per 10 μg/m^3^ (1.37–1.94) PM_10_: OR = 1.21 per 10 μg/m^3^ (0.87–1.55)
Cheng et al. (2022)	13 studies 1993–2018	Taiwan, Hong Kong, Italy, Sweden, the UK, and the US	36,430,147 individuals aged ≥50 years	PM_2.5_: HR = 1.47 per 10 μg/m^3^ (1.22–1.78)
Dhiman et al. (2022)	11 studies 1993–2013	Canada, Italy, Spain, Sweden, Taiwan, the UK, the US	9,655,613 individuals aged ≥50 years	PM_2.5_: HR = 1.08 per 1 μg/m^3^ (1.01–1.15) O_3_: HR = 1.02 per 1 μg/m^3^ (0.96–1.08)
Fu and Yung, 2020	9 studies 1993–2013	Canada, Italy, Spain, Sweden, Taiwan, the UK, and the US	11,603,390 individuals aged 30–85 years	PM_2.5_: OR = 1.95 per 10 μg/m^3^ (0.88–4.30) NO_2_: OR = 1.00 per 10 μg/m^3^ (0.89–1.13) O_3_: OR = 1.03 per 10 μg/m^3^ (0.68–1.57) PM_10_: OR = 1.32 per 10 μg/m^3^ (0.91–0.99)
Fu et al. (2019)	3 studies 2001–2010	Spain, Taiwan, and the US	686,486 individuals aged ≥60 years	PM_2.5_: OR = 3.26 per 10 μg/m^3^ (0.84–12.74)
Tsai et al. (2019)	3 studies 1999–2013	Taiwan, the UK, and the US	10,053,214 individuals aged ≥50 years	PM_2.5_: HR = 4.82 per 10 μg/m^3^ (2.28–7.36)
**Parkinson’s disease**				
Dhiman et al. (2023)	11 studies 2014–2019	Australia, Canada, Denmark, Netherlands, South Korea, Taiwan, and the US	7,236,528 individuals aged 35–85 years	PM_2.5_: 1.01 per 1 μg/m^3^ (0.99–1.03) NO_2_: OR = 1.01 per 1 μg/m^3^ (1.00–1.02) O_3_: OR = 1.01 per 1 ppb (1.00–1.02) CO: OR = 1.64 per 1 ppm (0.96–2.78)
Gong et al. (2023)	9 studies 2015–2022	Canada, Italy, South Korea, Taiwan, and the US	76,945,382 individuals	PM_2.5_: OR = 1.17 per 10 μg/m^3^ (1.00–1.33) PM_10_: OR = 1.00 per 10 μg/m^3^ (0.98–1.01)
Chen et al. (2022)	6 studies 1992–2013	Canada, China, Denmark, and the US	2,464,105 individuals	NO_2_: RR = 1.03 per 10 μg/m^3^ (1.01–1.05) O_3_: RR = 1.07 per 10 μg/m^3^ (1.02–1.12) CO: RR = 1.57 per 1 mg/m^3^ (1.07–2.32)
Han et al. (2020)	20 studies 1986–2018	Australia, Canada, China, Denmark, Germany, Greece, Italy, Japan, Netherlands, Spain, Sweden, Taiwan, the UK, and the US	16,078,950 individuals with average age of 71.3 years	PM_2.5_: RR = 1.08 per 10 μg/m^3^ (0.98–1.19) NO_2_: RR = 1.03 per 10 μg/m^3^ (0.99–1.07) O_3_: RR = 1.01 per 10 μg/m^3^ (0.82–2.11) PM_10_: RR = 0.99 per 10 μg/m^3^ (0.97–1.01) CO: RR = 1.32 per 1 mg/m^3^ (0.82–2.11)
Hu et al. (2019)	10 studies 1988–2013	Canada, Denmark, Taiwan, and the US	10,252,302 individuals aged 12–92 years	NO_x_: RR = 1.06 per 10 ppb (1.04–1.09) NO_2_: RR = 1.01 per 1 ppb (1.00–1.03) O_3_: RR = 1.01 per 1 ppb (1.00–1.02) CO: RR = 1.65 per 1 ppm (1.10–2.48)
Kasdagli et al. (2019)	13 studies 2007–2018	Canada, Denmark, Italy, Netherlands, Taiwan, and the US	15,576,835 individuals	PM_2.5_: RR = 1.06 per 10 μg/m^3^ (0.99–1.14) NO_2_: RR = 1.01 per 10 μg/m^3^ (0.98–1.03) O_3_: RR = 1.01 per 5 ppb (1.00–1.02) CO: RR = 1.34 per 1 mg/m^3^ (0.85–2.10)
Fu et al. (2019)	7 studies 1988–2010	Canada and the US	1,092,560 individuals aged 20–85 years	PM_2.5_: OR = 1.34 per 10 μg/m^3^ (1.04–1.73)

## Data Availability

No data was used for the research described in the article.
